# WOMEN’S STORIES: NARRATIVES ABOUT RELATIONSHIPS AND RESILIENCE

**DOI:** 10.1093/geroni/igad104.1734

**Published:** 2023-12-21

**Authors:** Wendy Watson, Sandra Faulkner, Charlie Stelle

**Affiliations:** Bowling Green State University, Bowling Green, Ohio, United States; Bowling Green State University, Bowling Green, Ohio, United States; Bowling Green State University, Bowling Green, Ohio, United States

## Abstract

Research has demonstrated that relationships are important in the lives of older women. Thirty-one women between the ages of 64-86 participated in interviews about their lives and relationships across the life course. In the interviews, women talked about their family of origin, friendships, romantic relationships, and their children and grandchildren. They explained how their relationships developed across the life course and how they have maintained their relationships, including how COVID-19 affected their maintenance behaviors. In the interviews, women constructed narratives that explained who they are as individuals and who they are in relationships. Through a poetic analysis of the interviews, resilience stood out as an overarching theme across the stories from women of different ages, marital statuses, economic backgrounds, and current and former occupations. Many women spoke of growing up in poor, rural areas and finding the strength to build a better life for themselves. There were stories of abuse, both in their family of origin and in chosen partners. Narratives included many instances of loss and hardships through caregiving, deaths of friends and relatives, and dissolution of relationships. Throughout it all, these women claimed resilience brought about through their own strength and determination and through relationships with people who supported them, mentored them, and built them up. This paper will discuss the many ways in which these thirty-one women exemplified resilience in their past, in their current lives, and as they looked toward their futures.

